# P2Y_12_ Inhibitor Pre-Treatment in Non-ST-Elevation Acute Coronary Syndrome: A Decision-Analytic Model

**DOI:** 10.3390/jcm5080072

**Published:** 2016-08-17

**Authors:** James Gunton, Trent Hartshorne, Jeremy Langrish, Anthony Chuang, Derek Chew

**Affiliations:** 1 Cardiology Department, Flinders University/Southern Adelaide Local Health Network, Adelaide 5042, Australia; jgunton@gmail.com (J.G.); trentharts@hotmail.com (T.H.); jeremylangrish@gmail.com (J.L.); Anthonychuang4@gmail.com (A.C.); 2 Oxford Heart Centre, Oxford University Hospitals NHS Trust, John Radcliffe Hospital, Oxford OX3 9DU, UK

**Keywords:** non-ST segment myocardial infarction, percutaneous coronary intervention, cardiac catheterization and angiography

## Abstract

Current guidelines recommend initiation of a P2Y_12_ inhibitor for all patients with non-ST-elevation acute coronary syndrome (NSTE-ACS) at the time of diagnosis (pre-treatment); however, there are no randomized trials directly comparing pre-treatment with initiation at the time of angiography to support this practice. We explore clinical and institutional parameters potentially associated with benefit with this strategy in a decision-analytic model based on available evidence from randomised trials. A decision analysis model was constructed comparing three P2Y_12_ inhibitors in addition to aspirin in patients with NSTE-ACS. Based on clinical trial data, the cumulative probability of 30 day mortality, myocardial infarction (MI) and major bleeding were determined, and used to calculate the net clinical benefit (NCB) with and without pre-treatment. Sensitivity analysis was performed to assess the relationship between NCB and baseline ischemic risk, bleeding risk, time to angiography and local surgical revascularization rates. Pre-treatment with ticagrelor and clopidogrel was associated with a greater than 50% likelihood of providing a >1% increase in 30 day NCB when baseline estimated ischemic risk exceeds 11% and 14%, respectively. Prasugrel pre-treatment did not achieve a greater than 50% probability of an increase in NCB regardless of baseline ischemic risk. Institutional surgical revascularization rates and time to coronary angiography did not correlate with the likelihood of benefit from P2Y_12_ pre-treatment. In conclusion, pre-treatment with P2Y_12_ inhibition is unlikely to be beneficial to the majority of patients presenting with NSTE-ACS. A tailored assessment of each patient’s individual ischemic and bleeding risk may identify those likely to benefit.

## 1. Introduction

Pre-treatment with anti-platelet agents, the co-administration of aspirin and a second agent such as P2Y_12_ or glycoprotein IIb/IIIa inhibitors prior to coronary angiography in patients with acute coronary syndromes without ST elevation, remains a complex and controversial topic in the current era [1,2]. The rationale for pre-treatment includes decreasing ischemic events prior to coronary angiography and a possible reduction in the risk of distal embolization and stent thrombosis during PCI by allowing adequate time for greater platelet inhibition to occur prior to balloon-injury [3,4,5]. However, pre-treatment may increase bleeding risk prior to angiography, at the time of coronary angiography during arterial puncture and sheath removal, and during cardiac surgery [3,6].

Withholding a second anti-platelet agent until the coronary anatomy is known may attenuate the risk of bleeding among those ultimately receiving cardiac surgery; however, it does not afford the ischemic protection of these agents prior to coronary angiography and revascularization. Furthermore, withholding pre-treatment prevents patients with an alternative diagnosis being over-treated and exposing them to increased bleeding risks without any potential for ischemic protection.

The net benefit of pre-treatment is therefore a balance between a reduction in ischemic events versus an increase in bleeding complications. This benefit may be modulated by specific patient factors, such as individual ischemic and bleeding risk, as well as institutional factors. The factors include the time delay to coronary angiography, which may increase the risk of ischemic events prior to revascularization, and local propensity for coronary artery bypass grafting with associated bleeding risks.

Many of the randomised clinical trials examining the efficacy of pre-treatment with P2Y_12_ inhibition are heterogeneous in their study populations, including patients with stable angina [7], not scheduled for angiography [5], randomised after angiography [8], post fibrinolysis [9], and only with ST-elevation acute coronary syndrome (STE-ACS) [10]. The ‘Comparison of Prasugrel at the Time of Percutaneous Coronary Intervention (PCI) or as Pre-treatment at the Time of Diagnosis in Patients with Non-ST Elevation Myocardial Infarction’ (ACCOAST) study is the only contemporary randomised trial of the timing of administration of a second antiplatelet agent in patients with non-ST elevation acute coronary syndromes [11]. The ACCOAST trial observed no ischemic benefit of pre-treatment with prasugrel; however, an increase in major bleeding complications was demonstrated [11].

Due to the lack of randomised clinical trial evidence, current European Society of Cardiology (ESC) guidelines are unable to recommend for or against pre-treatment with a P2Y_12_ inhibitor (in addition to aspirin) for patients with non-ST-elevation acute coronary syndrome (NSTE-ACS) [12]. Given the evidence gap in this area, we applied a decision-analytic model based on the available evidence from randomised trials to explore the possible clinical and institutional factors that may be associated with greater benefit with a pre-treatment strategy.

## 2. Experimental Section

### 2.1. Model Assumptions and Data Sources

**Figure 1:**
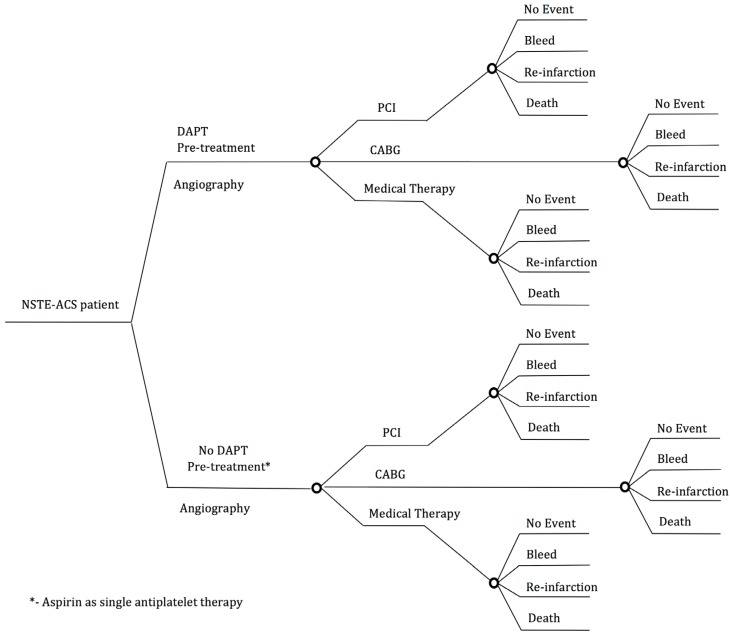
Decision tree representing the outcomes in the Monte Carlo model during the 30 day cycle. DAPT = Dual anti-platelet therapy, CABG = Coronary artery bypass graft, PCI = Percutaneous coronary intervention.

We developed a decision-analytic model to explore the impact of pre-treatment with P2Y_12_ inhibitors administered at the time of initial NSTE-ACS diagnosis compared with administration at the time of PCI in patients treated with a planned invasive management strategy. The model examined the effects of death or myocardial infarction (MI), and major bleeding, as well as net clinical benefit (i.e., combined death or MI and major bleeding events) within 30 days (Figure 1). Outcomes of the model included the ischemic outcomes of death or recurrent myocardial infarction as a summation of the estimated events prior to angiography, and then for each of the three treatment strategies (i.e., PCI, CABG and medical management) up to 30 days, with bleeding events before and after angiography similarly summated. To estimate the overall benefit of the strategy, we also report the net benefit of pre-treatment with each of the P2Y_12_ inhibitors, calculated as the absolute reduction in a 30 day ischemic event rate minus the absolute increase in bleeding event rate, (i.e., net clinical benefit (NCB)) between the pre-treatment and non-pre-treatment groups. For the purposes of this analysis, a clinically significant benefit was defined as a ≥1% increase in NCB at 30 days. The clinical relevance of this can be conceptualized as a Number Needed to Treat [NNT] of less than or equal to 100.

### 2.2. Baseline Assumptions of Patient Risk

Within this model, the baseline of death or MI within 30 days of diagnosis was set at 7% [13,14]. Half of this risk was experienced within the first seven days, consistent with early trials of NSTE-ACS where very early angiography (within 48 h) was not commonplace [5]. The risk of a major bleeding event within 30 days was estimated to be 2.5%, again with half of the risks experienced within the first seven days [13,14]. These risks were converted to hourly risks in order to allow separate estimations for event rates before and after angiography.

### 2.3. Procedural Rates, Timing and Impact on Risk

The median time to angiography was set at 24 h, with the proportion of patients selected for surgical revascularization (CABG) initially set at 10%, and the medical management without PCI set at 30%, with the remainder of the patients undergoing PCI, reflecting rates observed in local registries [13,14]. Revascularization was assumed to occur without subsequent delay and any associated ischemic and bleeding events occurring between angiography and revascularization with each strategy was included in the risk associated with that strategy. Among patients undergoing CABG, a relative risk reduction of death or MI by 30 days of 40% but a four-fold increase in major bleeding events compared to medical management was assumed. Similarly, for patients undergoing PCI, a 30% relative risk reduction in death or MI at 30 days, with a 1.25-fold increase in major bleeding, was assumed.

### 2.4. Modification of Risks Associated with Initiation of P2Y_12_ Inhibition

The relative effect of clopidogrel pre-treatment versus placebo, ticagrelor versus clopidogrel, and prasugrel versus clopidogrel were drawn from the results of the key large-scale randomized trials or subsequent published subgroup analyses where available [3,4,11,15]. For base-case analysis, estimated relative effects of death or MI, and major bleeding used the overall randomized trial findings, since these provide the best estimates for treatment-related effects of each anti-platelet agent since they are not subject to the usual limitations of subgroup analyses that apply to post-randomization sub-groups (Table 1). When examining the effect of prasugrel, we use the results of ACCOAST in the base-case since this was the only trial directly examining this question despite the fact that the TRITON TIMI 38 study was a much larger trial [3].

### 2.5. Sensitivity Analysis

To provide estimates of potential risks and benefits, 10,000 runs of the model were undertaken with bleeding events, ischemic events, and the NCB for each agent was calculated for each run. The proportion of scenarios where the net clinical benefit or net mortality benefit was positive relative to the comparator for each of the literature-based therapy comparisons (clopidogrel versus placebo, prasugrel versus clopidogrel, and ticagrelor versus clopidogrel) was summed and reported as a percentage. These estimates were then used to calculate the relative effect of ticagrelor and prasugrel over placebo and reported similarly. In order to quantify the magnitude of potential benefit, the absolute differences in the overall 30 day rate of ischemic events and bleeding events associated with pre-treatment compared with no pre-treatment were used to calculate the number needed to treat (NNT) for each run of the model.

The treatment effects associated with each of the P2Y_12_ inhibitors was allowed to vary across the range of the reported 95% confidence bounds for both death or MI and major bleeding. However, due to the differences in reported bleeding events associated with both ticagrelor and prasugrel, the bleeding risk associated with the proportion of patients undergoing CABG were modeled separately using relative hazards that differed from the overall population. For all of these analyses, P2Y_12_ inhibition was considered not to have any effect on peri-operative ischemic events.

To explore the relationship between varying degrees of baseline ischemic and bleeding risk, time to angiography, institutional rates of CABG and medical management, and treatment effects associated with the specific therapies, multi-way sensitivity analysis was employed. Within each iteration of the model, baseline ischemic risk was allowed to randomly vary between 0% and 15%, while the risk of major bleeding was varied between 0% and 6% with 70% of this variation linked to the ischemic risk given the strong correlation between ischemic and bleeding risks observed among individual patients clinically. Again, with half the 30 day risk experienced within the first seven days, both the bleeding and ischemic risks were converted to an hourly risk.

The time to angiography was allowed to vary randomly between 0 and 96 h, while the proportion of patients deemed appropriate for CABG and medical management were varied between 7% and 15%, and 35%–45%, respectively. However, recognizing that patients with an increased ischemic risk were more likely to undergo CABG rather than PCI, the likelihood of CABG was adjusted by a factor of 1.25 in those with an increased ischemic risk and 0.5 for those models with an ischemic risk of <5%. Therefore, a CABG rate of 10% was increased to 12.5% if ischemic risk was >7%.

The relationship between the likely NCB rates and the estimates of the NNT with varying degrees of: ischemic risk; bleeding risk; time to angiography; and the local surgical revascularization rate were explored. The number needed to harm (NNH) was calculated as 100/absolute event increase %. All models were constructed in STATA 14.0 (Manufacturer, College Station, TX, USA).

## 3. Results

A routine pre-treatment strategy was modelled, and the outcomes can be seen in Table 2. Overall, in this base case, there was no net clinical benefit for pre-treatment with any P2Y_12_ inhibitor with NNH varying from 22 to 500 with prasugrel and ticagrelor respectively.

### 3.1. Sensitivity Analysis

A one- and two-way sensitivity analyses was performed for each of the following variables assuming a range of plausible values: (1) estimated ischemic risk; (2) estimated major bleeding risk; (3) institutional CABG rate and (4) time to angiography.

### 3.2. Increase in Net Clinical Benefit (NCB) with Pre-Treatment

**Figure 2:**
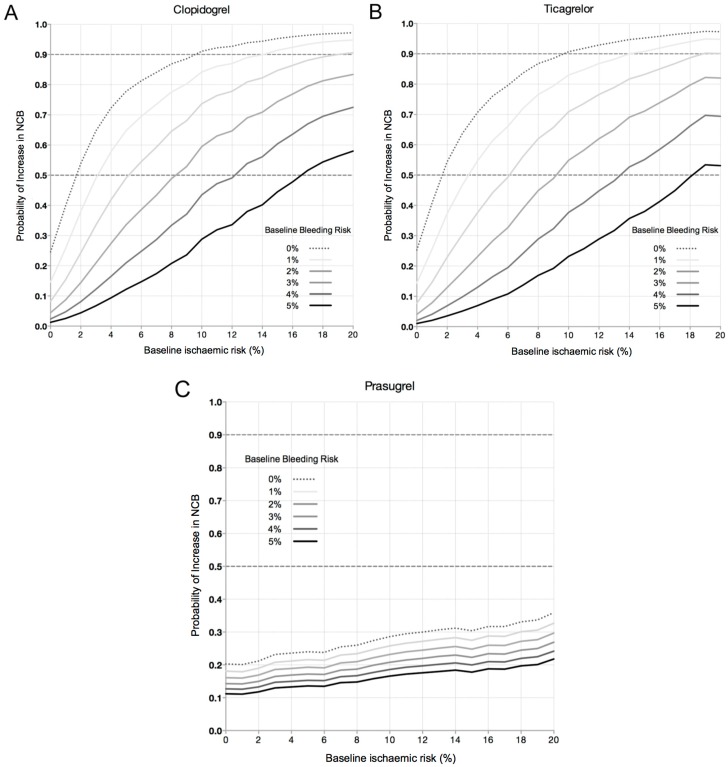
Estimated probability of achieving a positive 30 day Net Clinical Benefit (NCB) from pre-treatment with (**A**) clopidogrel; (**B**) ticagrelor and (**C**) prasugrel at varying levels of baseline ischemic and bleeding risk.

Pre-treatment with P2Y_12_ inhibition had a variable likelihood of achieving a positive net clinical benefits (NCB) depending on the agent used and the patient’s level ischemic and bleeding risk. Figure 2A–C display the likelihoods of achieving a positive NCB for each of the three anti-platelets agents at varying levels of ischemic risk, with 30 day bleeding risk plotted for values between 0% and 5%. With all three agents, increasing levels of ischemic risk resulted in a higher likelihood of achieving a positive NCB. However, the likelihood was strongly modulated by bleeding risk. As seen with clopidogrel, patients with a baseline ischemic risk of 6% and a 30 day bleeding risk of 0% had an 82% likelihood of achieving a positive NCB. However, when the 30 day bleeding risk increased to 5%, those with a 6% baseline ischemic risk had only a 15% likelihood of achieving a positive NCB. A similar trend was seen with ticagrelor, where patients with a 5% bleeding risk only reached a 50% likelihood of achieving a positive NCB at a baseline ischemic risk of 18%.

Importantly, a greater than 50% likelihood of achieving a positive NCB was only reached with clopidogrel and ticagrelor. Prasugrel was associated with low likelihoods of achieving a positive NCB, at all levels of ischemic and bleeding risk. Even using estimates of efficacy and safety from the TRITON TIMI 38 study for prasugrel suggests low likelihoods of a positive NCB due to the higher rates of bleeding with this agent (data not shown).

**Figure 3:**
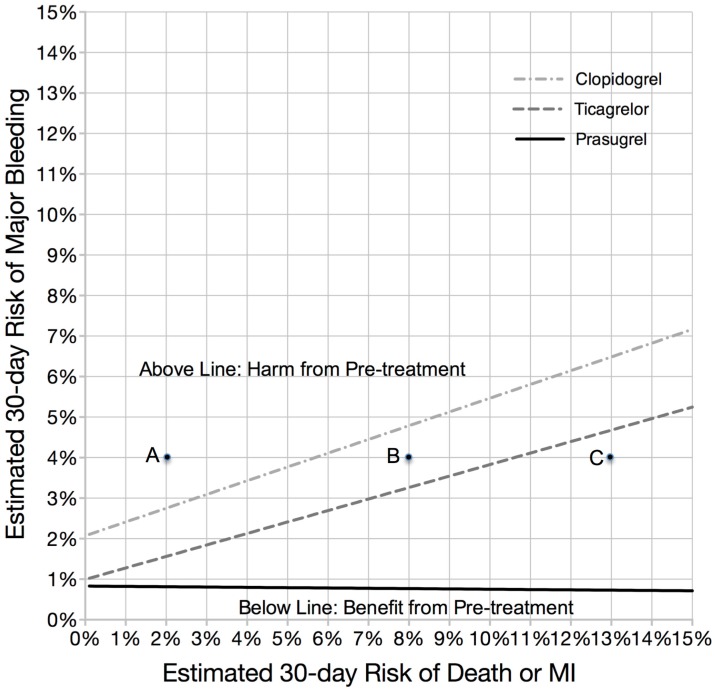
Frontier plot with each line representing the estimates of 30 day Net Clinical Benefit at varying levels of baseline ischemic and bleeding risk for clopidogrel, ticagrelor and prasugrel. Area below each line indicates a neutral or positive net clinical benefit from pre-treatment. Using this plot and assuming a 4% 30 day risk of major bleeding, pre-treatment is harmful with any P2Y_12_ inhibitor at 2% 30 day ischemic risk (Point A), beneficial with clopidogrel only at 8% 30 day ischemic risk (Point B), and beneficial with both ticagrelor and clopidogrel at 13% 30 day ischemic risk (Point C).

Figure 3 further describes the relationship between achieving a positive NCB from pre-treatment based on a patient’s estimated 30 day risk of death or myocardial infarction (ischemic risk) compared with their estimated 30 day risk of major bleeding (bleeding risk). Points below the line delineate baseline ischemic and bleeding profiles being associated with a 50% or more likelihood of achieving a positive NCB with pre-treatment. At low levels of ischemic risk, both agents were associated with benefits where the bleeding risk remained very low. However, as ischemic risk increased, a benefit was only found if the estimated bleeding risk remained below the line of intersection for each agent. Due to the greater ischemic benefit associated with ticagrelor, a greater tolerance for baseline bleeding risk is observed. With regards to prasugrel, harm from treatment was found with all levels of estimated 30 day ischemic risk, except in patients with the lowest bleeding risk (estimated 30 day bleeding risk <1%).

### 3.3. Time to Coronary Angiography and Institutional CABG Rate

There was no correlation between the time to angiography and benefits with a strategy of pre-treatment with any of the three P2Y_12_ regardless of a patient’s ischemic and bleeding risk.

Overall, variability in the institutional CABG rate had a minimal effect on providing an overall benefit with pre-treatment with each of the three agents.

### 3.4. Magnitude of Effect: The Likelihood Providing a Significant Clinical Benefit from Pre-Treatment

**Figure 4:**
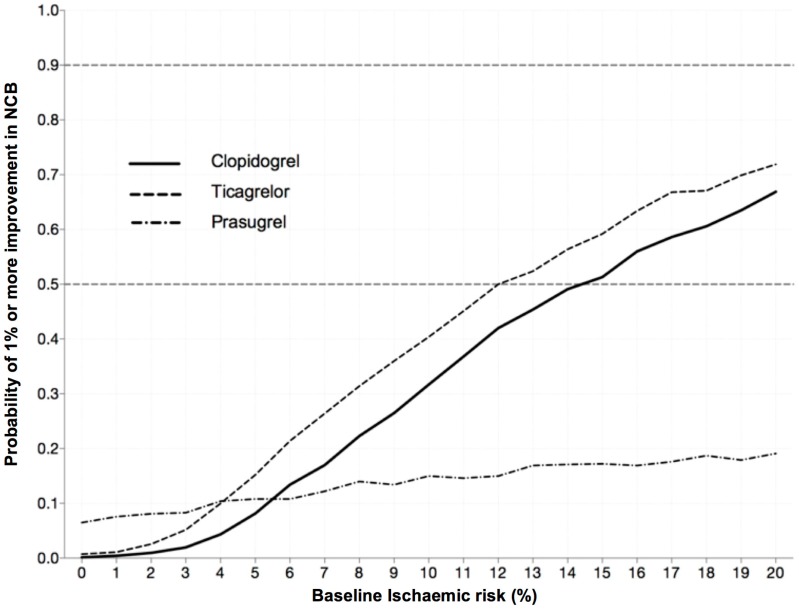
Estimated likelihood of deriving a significant clinical benefit from pre-treatment with clopidogrel, ticagrelor and prasugrel at varying levels of baseline ischemic risk. Significant clinically benefit defined as absolute increase in 30 day Net Clinical Benefit by 1% or more (NNT < 100).

As patients’ ischemic risk increased, there was a higher likelihood that pre-treatment with either ticagrelor and clopidogrel would achieve a significant clinical benefit defined as a 1% increase in NCB at 30 day (Figure 4). Hence, pre-treatment with clopidogrel in a patient with a 30 day ischemic risk of 3% had less than 3% likelihood of a achieving a significant clinical benefit. This likelihood rose to 60% among those with a 30 day ischemic risk of 18%. Similarly, with ticagrelor, a patient with a 30 day ischemic risk of 3% had only approximately 5% likelihood of providing at least a 1% increase in NCB; however, this increases to 68% with an ischemic risk of 18%. Pre-treatment with prasugrel was associated with very low likelihoods of achieving significant clinical benefits, even in patients with a high ischemic risk.

## 4. Discussion

Major society guidelines either support the use of pre-treatment in patients with acute coronary syndromes or refrain from advising on pre-treatment due to the lack of support from clinical trial data [12,16]. ACCOAST, a contemporary randomized clinical trial assessing prasugrel pre-treatment in NSTE-ACS, was unable to demonstrate a reduction of ischemic outcomes [11]. We built a decision analysis model for the clinical utility of pre-treatment based on the relative risk reductions from major clinical trials on the currently recommended dual anti-platelet agents [5,7,8,9,11]. The likelihood of providing a substantial benefit with pre-treatment (NNT < 100) was not observed unless the baseline ischemic risk exceeded 11% and 14% for ticagrelor and clopidogrel, respectively. It should be noted that these estimates are sensitive to the baseline risk of bleeding, and since clopidogrel was modelled with less impact on bleeding, this allows for a greater tolerance of baseline bleeding risk using this agent. Institutional characteristics of time to angiography were not associated with increased net clinical benefit and institutional CABG rates had only minimal effects on net clinical benefit with pre-treatment using each of the agents. This analysis suggests that a pre-treatment strategy with P2Y_12_ inhibitors is confined only to patients at relatively high predicted ischemic risk. Consequently, decisions to initiate pre-treatment should be dependent on careful and potentially objective evaluation of ischemic and bleeding risk. This can be done easily at the patient bedside with well-validated risk scores such as the Global Registry of Acute Coronary Events (GRACE) score.

Large-scale randomized studies of intravenous glycoprotein IIb/IIIa inhibition given “upstream” before coronary angiography in NSTE-ACS have failed to demonstrate a significant reduction in ischemic events when compared with these agents used in the peri-procedural setting [17,18]. Pre-treatment with P2Y_12_ inhibition in NSTE-ACS remains controversial because much of the evidence supporting its practice is derived from trials which included patients with stable angina, STE-ACS, and those having received fibrinolysis for STE-ACS [7,8,9,10,11]. Data from small observational studies suggest reductions in peri-procedural ischemic events associated with pre-treatment with clopidogrel, with similar incremental benefits observed with large loading doses compared with small loading doses of this agent, but these analyses are confined to those actually receiving PCI [7,19,20,21,22]. In contrast, the first randomized comparison of this question, Clopidogrel for the Reduction of Events during Observations (CREDO) study [8] included patients who were planned for PCI (and therefore included elective patients), and the PerCutaneous Intervention-CLopidogrel as Adjunctive ReperfusIon TherapY (PCI-CLARITY) trial [9] included only those with ST elevation who had received thrombolysis. A sub-study of the large Clopidogrel in Unstable angina to prevent Recurrent Events (CURE) trial [15] reported a median time from randomisation to angiography was 10 days, making direct comparisons to the modern era where time to angiography is generally less than 48 h, less reliable. However, the patient population in CURE only included those with acute coronary syndromes without ST elevation, and therefore represented the specific group of interest for this discussion [5].

Several meta-analyses attempting to address this question have included these studies, and have reported a benefit of pre-treatment with clopidogrel in PCI patients [20,23,24]. This was demonstrated in the 2012 meta-analysis of 37,814 patients by the Action group, which showed a statistically significant reduction in major cardiac events (9.83% versus 12.35% *p* < 0.001) in those pre-treated with clopidogrel [20]. This study also found no significant difference in mortality with pre-treatment and no significant association between pre-treatment and major bleeding [20]. However, these studies have focused on the subgroup of patients who actually received PCI, and excluded those who underwent coronary artery bypass grafting or continued medical management. Hence, these meta-analyses focus on the group of patients who are likely to have the most favourable balance between ischemic benefit and bleeding risk. In contrast, a meta-analysis of patients undergoing coronary artery bypass grafting from both observational and randomized studies demonstrates a clear increase in bleeding events with an increase in mortality among these patients [6]. In a meta-analysis of three randomised trials and 17 observational studies, Biancari et al. demonstrated that pre-operative exposure to clopidogrel was associated with an increased risk of death (RR: 1.3; CI: 1.02–1.67), re-operation for bleeding (RR: 1.88; CI: 1.37–2.58) and need for packed red blood cells (RR: 1.23; CI: 1.10–1.37) [6].

ACCOAST is the only other randomized control trial of pre-treatment in ACS patients without ST elevation. This study compared a half load of prasugrel as pre-treatment with a further half dose after the coronary anatomy had been identified by angiography versus 60 mg of prasugrel post-angiography among those undergoing PCI, with mean time from pre-treatment to coronary angiogram times of 4 h [11]. PCI was performed in 69% of patients, CABG in 6.2%, and medical management in 25%. Pre-treatment did not reduce ischemic outcomes of NSTE-ACS; however, it resulted in increased bleeding events [11]. As a result of this trial, the ESC guidelines caution against pre-treatment with prasugrel; however, they are unable to advise for or against pre-treatment with other P2Y_12_ inhibition in the management of NSTE-ACS [12]. It is worth mentioning that ticagrelor pre-treatment was studied in the randomized control trial, “Administration of Ticagrelor in the Cath Lab or in the Ambulance for New ST Elevation Myocardial Infarction to Open the Coronary Artery” (ATLANTIC) among patients with STE-ACS, and resulted in no reduction in the composite end point of death, myocardial infarction, stroke, urgent revascularization or stent thrombosis, though a very short time difference in dosing times between the two arms (i.e., 31 min) occurred in this study [10].

Based on our analysis, pre-treatment with ticagrelor or clopidogrel is anticipated to be of benefit in specific populations of patients presenting with NSTE-ACS, particularly those with a high risk of ischemic events. Pre-treatment with ticagrelor and clopidogrel could result in a greater than 50% probability of achieving a positive 30 day NCB at lower levels of individual bleeding risk combined with higher baseline ischemic risk. This emphasises the need for the clinician to be able to accurately identify a patient’s ischemic risk using well-validated tools such as the GRACE risk scoring system. For example, a GRACE score of approximately 150 translates to an ischemic risk of >11%, representing the threshold for ticagrelor pretreatment [25]. Hence, while the validity of internationally derived risk scores requires local calibration, this risk-score threshold based decision is analogous to using the CHA2DS2-VASc score for identifying patients at sufficient stroke risk warranting anticoagulation in non-valvular AF. Nevertheless, prospective confirmation of pretreatment thresholds is desirable, and future studies of pretreatment should clearly stratify patients by baseline ischemic and bleeding risk.

Given the greater levels of platelet inhibition, ticagrelor was associated with an increase in NCB at lower thresholds of ischemic risk than observed with clopidogrel. However, among patients with the highest bleeding rates (5%), ticagrelor pre-treatment was only beneficial when the ischemic risk exceeded 18%. Conversely, in the same high risk bleeding population, clopidogrel pre-treatment required an ischemic risk of only 16% in order to obtain a greater than 50% probability of achieving a positive 30 day NCB.

### Limitations

Given the paucity of clinical trial data specifically addressing the question of pre-treatment in patients with NSTE-ACS, this decision-making analysis relies upon assumptions from numerous clinical trials. Firstly, the initial assumptions for clopidogrel are based on the CURE trial, which reflected clinical practice of over a decade ago, before the introduction of radial arterial access, longer delays to angiography, less refined coronary stent technology and less adjunctive anti-ischemic therapies available [5]. The net effect of the aforementioned differences may in fact be an overestimation of the ischemic benefit of pre-treatment, as suggested in a recent meta-analysis in which a modest effect of major cardiovascular events was noted in older randomized control trials such as CURE, but was not found in more recent studies [5,23]. Hence, our analysis may well over estimate the benefits of pre-treatment with clopidogrel and then, consequently, the newer P2Y_12_ agents. For the purposes of the decision-making analysis, the model assumes therapeutic efficacy at the time of administration of the P2Y_12_ inhibitor. In the clinical setting, there would be a delay prior to peak therapeutic efficacy with the time dependent on the P2Y_12_ inhibitor employed.

Secondary, the pre-treatment effects of ticagrelor were modelled on the PLATO trial, which did not formally examine pre-treatment of this agent [4]. Nevertheless, it offers the best available evidence for the pre-angiography use of ticagrelor compared with clopidogrel in the NSTE-ACS population.

This analysis does not take into account patients with presumed NSTE-ACS who go on to have normal coronaries with an alternative diagnosis such as myocarditis. Given these patients do not proceed to PCI or CABG, the absolute effect on their ischemic benefit or bleeding complication rate is likely to be negligible. Again, as a consequence, actual benefits of pre-treatment in real world practice may be less striking than what is presented in this decision-analytic model.

## 5. Conclusions

Decision analysis using treatment effects observed in randomized clinical trials, across plausible ranges of ischemic and bleeding risk suggests that pre-treatment may only benefit patients with NSTE-ACS with a very high ischemic risk, and the practice of routine use may be harmful. The magnitude of this benefit is likely to benefit those in the subgroups of patients at a low risk of bleeding and is likely to be small. Further analysis with an appropriately powered randomised control trial would help clarify this issue but will be challenging given the very small effect expected from pre-treatment. This decision- analysis model is not sufficient to warrant a change in current guidelines for the management of NSTE-ACS; however, it highlights the need for controlled trials assessing the benefits of pre-treatment with P2Y_12_ inhibitors, ticagrelor and clopidogrel, with randomization stratified by objective measures of ischaemic and bleeding risk.

## Figures and Tables

**Table 1:** Base case values used for decision analytic model on NSTE-ACS pretreatment.

Base Case	Value (Confidence Interval)	Reference
Baseline ischemic risk	7%	[13,14]
Baseline bleeding risk	2.5%	[13,14]
Delay to angiography	0–96 h	
CABG rate	7%–15%	
Clopidogrel effect on death/myocardial infarction rates	0.80 (0.72–0.90)	[15]
Clopidogrel effect on bleeding rates	1.38 (1.13–1.67)	[15]
Prasugrel vs. Clopidogrel on death/myocardial infarction rates	0.98 (0.78–1.23)	[11]
	0.81 (0.73-0.91)	[3]
Prasugrel vs. Clopidogrel on bleeding rates	2.86 (1.44–5.68)	[11]
	1.32 (1.03–1.68)	[3]
Ticagrelor vs. Clopidogrel on death/myocardial infarction rates	0.83 (0.74–0.93)	[4]
Ticagrelor vs. Clopidogrel on bleeding rates	1.03 (0.93–1.15)	[4]

**Table 2:** Base case modeled with an untreated baseline ischemic risk of 7% and a bleeding risk of 2.5%.

No Pre-Treatment (at 30 Days)		P2Y_12_ Inhibitor	Pre-Treatment Risk (at 30 Days)					
Death or MI	Major Bleeding		Death or MI	Major Bleeding	ARR (Death or MI)	ARI (Major Bleeding)	NCB	NNH
6.3%	3.5%	Clopidogrel	5.1%	4.9%	1.2%	1.4%	−0.2%	500
		Prasugrel	5.1%	9.2%	1.2%	5.7%	−4.5%	22
		Ticagrelor	4.2%	6.8%	2.1%	3.3%	−1.2%	83

ARR: Absolute risk reduction; ARI: Absolute risk increase; NNH: Number needed to harm; NCB: Net clinical benefit (calculated as absolute risk reduction in ischemic events minus the absolute increase in bleeding event rates).

## Abbreviations

The following abbreviations are used in this manuscript:

DAPT	Dual anti-platelet therapy
CABG	Coronary artery bypass graft
PCI	Percutaneous coronary intervention
